# The “Horse Industry” in Kansas

**Published:** 1895-06

**Authors:** 


FROM the *Horse World* we quote from a paper read by F. H. Avery, of Wakefield, Kansas, on the "The Horse Industry." Referring to the future markets he cites the advent of the modern paving-stone, requiring probably two horses to do the same period of service that one did before.

"Under the impetus of irrigation immense areas of arid land are being brought rapidly to a high state of cultivation. The large farms of the country are being subdivided and subjected to a more thorough system of cultivation, all of which will call into requisition more horses. The importation of horses, even for breeding purposes, has practically ceased. We are beginning to export horses to European countries, and in time will, no doubt, build up an extensive export trade with these countries. Mexico and Central and South America, as in everything else, are beginning to look to us for breeding-stock with which to improve their native horses. The Pacific Islands are taking considerable numbers of our horses. The Southern States of our own country used to purchase but comparatively few horses in our Northern markets, but for the last few years they have been enjoying a period of remarkable growth and development, and, with other causes operating to assist, they now, probably, call for more horses annually than the street railways ever did.

"Now if, in the meantime, severe depression has overtaken the business and breeders have become discouraged and are sacrificing their brood-mares, and even forsaking the business, there is certain to be a marked decrease in the supply of horses. That there has been an umistakable falling off in the extent of breeding here in Kansas is a fact familiar to us all, and distinctly and emphatically so to stallion-owners. In my own section of the State the decrease is as great as seventy-five per cent., and I presume that this is not far from the average for the entire State. Kansas seems to be a pretty good index for the whole country. A year ago a prominent breeder of Illinois sent out a thousand letters of inquiry to all parts of the United States and Canada, and with the exception of about twenty-five they answered. 'The farmers in this vicinity are giving up breeding horses.' A commission dealer of Boston writes that he is handling large numbers of mares, from seven to nine years old, of excellent type. They have evidently been kept for brood-mares. He says that these and other circumstances lead horse-dealers there to the conclusion that there is going to be a short supply of horses at a not very distant date. Briefly stated, then, the conditions are these: We have a decreased supply to meet a constant demand with, and the effect, other things being equal, will be an increase in prices. But the change may not become perceptible for a year or longer, and we may scarcely expect prices to get back to what they were once.

"Well, we have been taking municipal reform, legislative reform, and tariff reform in allopathic doses for some time, but the system of horse-breeding has been crying for reform in vain. In the palmy days of the industry the character of the market permitted the utmost latitude in management. Careless breeding, careless feeding, and careless preparation for market were the rules, and even then almost every kind of a horse found a buyer at a price generally remunerative. Certainly, even then, the more judgment exercised in breeding and the more care used in feeding and the better the preparation for market, proportionately greater were the returns, but, generally speaking, all classes of horses yielded a profit to the breeder. The prices of other stock fluctuated widely, leaving a balance sometimes on the right side of the account and often again on the wrong side, but the prices of horses remained uniformly high and the balance steadily on the right side of the account. People began to believe that the demand for horses was a constantly increasing one and that the market could never be glutted, with the natural result that everybody wanted to get into the horse business. Men borrowed money and went into the business and others took it up as a kind of a genteel side-line. Well, the latter have been finding all that they could attend to in their legitimate pursuits of late; and most of the fellows that borrowed money and invested it in the business took it out in experience and paid their creditors in the same commodity.

In the early rush for grand-stand privileges, we selected sires from every known breed under the sun, with results equally as diversified. For mares to breed from we saved the good, bad, and indifferent. If they were balky or blemished or vicious, worthless to work, and impossible to sell, we saved them, just the same, to perpetuate their infirmities What are the results? The quality of the horses in Kansas is probably not above what it was five years ago. Another cause has helped to bring this about. There is a tendency all the time for a man to sell his good horses and keep his poor ones. Not that he wants to do so, but the buyer wants to buy them. The good ones are nearly always the fat ones, and the harder the times the more likely the owner is to part with them. The remedy is not to keep the inferior ones fat and the good ones poor, but to keep them all fat and keep the inferior ones in sight and the good ones out of sight.

"But to return to the matter of sires and dams. There is a whole lot of horses eating their heads off in Kansas this year that were sired by nothing in particular, but damned by everybody in general. Administer a dose of reform here. Begin a little ruthlessly, as the custom is. Weed out every undersized, blemished, or inferior animal and dispose of them. You probably will not get very rich from the proceeds, but it will, nevertheless, be a sagacious business transaction fraught with good and lasting results. While the reform movement is on do not spare some scrubby old mare because she raises a colt every year. Every colt she raises will run you in debt. Ordinarily these measures of reform would seem unnecessarily rigorous, but the exigencies fully demand them and the conditions are ripe for them. There will never be a more advantageous opportunity to replace cheap, inferior animals with good ones than the present time affords.

"Lastly, fix an ideal and keep it ever in view. Whether you are an admirer of trotting horses, do not mix them up with the hope of getting a general-purpose horse. Life is too short for ordinary horse-breeders to establish any more types of horses, and our posterity may take to the poultry business."

Mr. James Leithwood, F.R.C.V.S., in addressing the Cheshire Farmers' Club, of England, on tuberculosis, has this to say on the question of eating tuberculous meat:

1. The disease is undoubtedly the same in man and animals, and due to the same microbe.

2. Man is the most susceptible to the disease of all animals.

3. Animals much less susceptible to the disease than man become affected by eating the flesh of diseased animals.

4. The deadly virus, the tubercule bacillus, is found in the blood-stream and lymphatics, and hence is conveyed to all parts of the body.

5. Considerable disease and swarms of bacilli have frequently been found in the marrow of bones, and it is therefore impossible to remove all diseased parts when they cannot be seen.

6. The flesh has been proved by experiment to be infective; of thirty-five animals fed on raw flesh 22 per cent. became affected. In another experiment of forty-six fed in the same manner 13 per cent. suffered. Pigs similarly fed for ten days all became diseased (Peuch). Rabbits became affected from juice of muscles from a cow in very good condition, and muscle has been found visibly diseased by Sir C. Cameron, McCall, and others.

Ordinary cooking is insufficient to destroy infectivity; the juice from roasted pork was still able to infect rabbits. Tuberculous flesh after being in boiling water for fifteen minutes still infected 35 per cent. in sixty-two experiments. Tuberculous juice heated in 198° F. infected one animal in six.

In cooking meat the albumin coagulates at 162° F., and if the red juice oozes out when carving the albumin is not coagulated, which shows that the temperature has not been sufficient to kill the bacilli.

Kastner caused the disease in nine out of eleven cases with injection of muscle-juice. Professor Galtier has also produced the disease with the juice of muscle. Professor McFadyean says: "Every animal having localized tuberculosis is exposed to the risk of generalization by the bacilli gaining access to the blood-stream, and it is never possible to state with certainty that such a condition is not already in existence." He also asserts that he has frequently seen butchers when dressing carcasses wipe their knives across the flesh after using them for removing tuberculous lesions. I maintain that if flesh from graped animals must be sold for human food let it be labelled as such, and see who will buy it.

The public have a perfect right to be protected from deception in fresh meat just as much as in butter or any other commodity, and when they go to the shop to buy beef they ought to get beef, and not beef plus a deadly virus. There are many good men who are in doubt whether there is a risk in the consumption of tuberculous meat or not, but I contend that even they ought to give the human life, and not the microbe, the benefit of the doubt. When we lost 35,000 souls in the Crimean War was it not considered to be a great national calamity? And yet we serenely allow this terrible foe to take from us every year four times that number, and do next to nothing to stem the march of the enemy. I am quite of opinion that the quantity of bacilli in the muscles of the affected animals may not be large, that cooking the flesh undoubtedly destroys some of them, and that even though a few may remain it is probable that the flesh may be eaten with impunity by the whole population of this country, if everyone of them were in perfect and robust health.

But what of those who are not so? Are there not thousands of them? How many just recovering from some severe illness; how many debilitated from insufficient food and clothing; how many, from numberless causes, whose vitality has been brought below the powers of resistance to this deadly foe? There are thousands of these weakly ones always with us, and, as I have already told you, this is just the condition which is most favorable for the growth of the microbe. This organism, like every other parasite, either animal or vegetable, thrives best on and glories in the weakness of its host; and are we going to continue to allow this almost infinitesimal foe to have its own way, and to pick out from among us its yearly number of victims to the extent of 150,000 human lives?

I earnestly hope and honestly think we shall not, and I unhesitatingly affirm that not one ounce of flesh from any tuberculous animal, whether it be from cattle, horses, pigs, sheep, fowls, or rabbits, ought to be permitted to be used for human food, not yet even for the food of animals.

The milk of tuberculous animals is still more infected and still more dangerous than the meat (especially when the udder is affected) owing to its being generally used in its uncooked state. Professor Macqueen says: "No one can gauge the amount of mischief done through the use of milk that is too often loaded with the germ of tuberculosis." Professor Arloing says that the danger is so great to children that it can scarcely be exaggerated. Over 2000 tuberculous children under two years of age die annually in Paris alone, and it is probable that more than half of these are due to the consumption of tuberculous milk. Professor Delepime reports a case of a child, four months old, without any hereditary predisposition, dying from tuberculosis of the mesenteric glands. It had been fed with milk from a cow kept specially for the purpose, which, when killed, was markedly tuberculous and bacilli were found in the udder. Dr. Sims Woodhead says "mesenteric or abdominal tubercles are far the most frequent in children." Professor McFaydean and Dr. Woodhead found that tubercle of the intestine was present in 43 cases out of 127 analysed, and that the tubercle of the mesenteric glands was present in 79 per cent. of cases, and more than half of these were found in children between the age of one and five years, during the period after they were weaned, when cow's milk would be taken in the greatest quantity. From a series of inoculations with milk from tuberculous udders over 70 per cent. had become affected. Hirschberger finds that the bacilli are found in the milk in 33 per cent. of tuberculous cattle, and Ernst inoculated with milk from cows without udder affection and caused the disease in 37 per cent. of cases.

The bacillus has been found to retain its vitality for ten days in milk, fourteen days in whey and cheese, and thirty days in butter. Dr. Ferro, of Turin, has found 10 per cent. of different samples of foreign butter to contain the microbe and infect guinea-pigs fed with it.

There are numbers of cases to prove the dangerous power of infected milk, which is recognized by the most reliable authorities to be the chief source of tubercle in children in the form of tabes mesenterica and tubercular meningitis. All the great authorities are agreed on this point, that the question of the consumption of tuberculous milk is one of the greatest possible importance to the children of this country; and I am sure you will agree with me that it is the bounden duty of every one of us to do our utmost to protect these innocent and helpless ones who are incapable of doing anything themselves against this deadly enemy.

A striking instance of infection in a family is related by Mr. Tedbar Hopkin, in which all the females who drank milk became tubercular, while the male members of the family, whose favorite beverage was whiskey, remain healthy to this day. The only safeguard now practised against infected milk is to see that it is well boiled for not less than five minutes. There is also less risk in using the mixed milk from a large number of cows than from one single animal. If that one cow should be tubercular it is almost a certainty that a child continually fed from it must become affected.

Then, let us not, gentlemen, shirk our responsibility in the matter, but, like men, look straight at this great danger, and be not satisfied until we have removed every possibility of infection from our milk-supply, and thereby effectually clear away that element of doubt that now hangs over our best, cheapest, and most perfect of all foods. There is nothing that contains every element of nutrition in its proper quantities to nourish the body except pure milk; all other foods have either some element wanting or something in excess, and if the working population could be persuaded to use pure, nutritious milk instead of so much tea (which does not contain one atom of nourishment), it would add much to the strength of the future generation of this country.

Dr. S. G. Dixon, of the Academy of Natural Sciences, speaking on the subject of "Purity of Milk," before the Horticultural Society's Farm and Dairy Section of Philadelphia, said:

"Rarely, if ever, do the tubercle bacilli find their way into the milk from a consumptive cow, unless she has tuberculosis of the milk glands, and, in connection with this statement, I have reason to believe that only about $2\frac{2}{5}$ per cent. of the tuberculous cows have such mammary tuberculosis.

"If this be so, we should not allow onr attention to be diverted from other and more fertile sources from which the tubercle bacilli may find their way into the milk. For example, consider the probability that there may be a greater number of consumptive attendants about a dairy than of cows with tuberculous udders, since only $2\frac{2}{5}$ per cent. of all consumptive cows are in such condition, while nearly one-seventh of all human deaths occur from tuberculosis, and then picture to yourself an attendant, well advanced in pulmonary consumption, poverty-stricken, careless and uncleanly as regards his clothing and person, who not only scatters thousands upon thousands of the bacilli by expectoration upon the hay, bedding, and all about the stable and dairy, but even washes the germs from his hands, which he has failed to cleanse before milking, directly into the pail. Other ways by which the organisms might find their way into the milk before its delivery to the consumer will readily occur to you; but, while I fully appreciate the great importance of having milk from healthy animals, the above will suffice to show you why I do not believe in straining at gnats and swallowing camels.

"We do not want to kill every cow in Pennsylvania that has simply a localized pulmonary consumption, because few, if any, of these give tuberculous milk; but we do want to improve the health of our animals, and, above all things, to have as dairy attendants persons who are not suffering from consumption or other loathsome and dangerous diseases.

"The first step to be taken to guard against the impurities of which I have spoken will be to send to every farmer of our own and adjoining States printed circulars informing him of the sources of danger, and showing him how necessary it is for him to have his stables clean, well lighted, well ventilated, and dry; to keep the animals themselves clean and well fed with varied and nutritious food, and with access to plenty of pure water three times a day at least; to have only clean and healthy men and women about the stables and dairy; to have a wash-room with hot and cold water, plenty of soap and clean towels near the milking stables, etc. Then, if the average farmer is as much of a man as are those it is my pleasure to know, he will try to make use of this information, and will do much to protect the milk from the various germs of disease.

"No chemical or other substance should be added to the milk, either to color it or to render it alkaline. The vessels for keeping and conveying the milk to consumers should be thoroughly cleansed, scalded, and exposed to sunlight and air each time after use, and the separate jar system requires especial care in this respect, for the jars are frequently carried into sick-rooms, where children may be ill with scarlet fever or other infectious diseases, and may be returned to the milkman and thence to the dairy without having been properly sterilized. If the dairyman fails to sterilize it, it may serve to carry the infection to other families or children. This, however, is only a danger when the milkmen are ignorant and careless, and of such let us hope that there are few. I might here suggest the delivery of milk in jars so cheap that they could be destroyed or discarded immediately after use. I should think that such jars might be made and furnished to dairymen in quantity for a fraction of a cent a piece.

"Again, I think we should try to spread the information and to teach mothers and others that good milk is not necessarily of the rich yellow color such as they demand and tempt the dairymen to produce by the use of artificial coloring matters.

"Lastly, we should realize that if we want milk from a healthy herd of clean, well-fed cows, kept in well-lighted, well-ventilated, dry stables, attended by healthy, clean, and intelligent persons, we should realize, I say, that such milk cannot be furnished at the present time in Philadelphia for less than nine or ten cents a quart, and we should be willing to pay this price and to allow the producer to make something more than a starvation living from his business."

Tuberculous Meat.—It is now universally acknowledged that the flesh of animals suffering from the disease in a severe form, with fever and emaciation, ought to be absolutely condemned as unfit for human food, and ought not to be given to carnivorous animals, but destroyed.—*British Medical Journal.*

